# THE PARADOX OF DIGITAL CONNECTIVITY: COMPARING THEORIES ON LONELINESS AND MOBILE PHONE DEPENDENCE

**DOI:** 10.1093/geroni/igae098.3748

**Published:** 2024-12-31

**Authors:** Hailey Andrews, Nelson Roque

**Affiliations:** Pennsylvania State University, University Park, Pennsylvania, United States; Pennsylvania State University, University Park, Pennsylvania, United States

## Abstract

In the Digital Age, smartphones and tablets are widespread, but their impact on well-being, particularly the relationship between loneliness and mobile phone dependence (nomophobia), remains debated. The direction of this relationship is unclear, with limited research across the lifespan. This study examines two theories: the Displacement Hypothesis, which suggests that nomophobia leads to loneliness, and the Theory of Compensatory Internet Use (CIUT), which proposes that loneliness drives nomophobia. Data were collected from 550 adults (Mage = 40.15, SD = 12.23, 54% female; 59% non-Hispanic White) via an online survey, delivered on Prolific. Linear regression models, adjusting for sociodemographics (age, gender), evaluated the relationship between measures of mobile phone dependence (Nomophobia Questionnaire) and loneliness (UCLA Loneliness Scale). For the model with loneliness as an outcome, (N = 550), age (β = -0.10, p =.028) and nomophobia (β = 0.06, p =.003) were significant predictors. For the model with nomophobia as an outcome, (N = 550), age (β = -0.35, p <.001), gender (β = -6.40, p =.009), and loneliness (β = 0.28, p =.003) were significant predictors. Both directions showed a significant relationship, however, the model with loneliness as a predictor of nomophobia explained slightly more variance (4.6%) in the outcome (vs 2.5% for nomophobia predicting loneliness). Results suggest that reducing loneliness could help mitigate mobile phone dependence. Future longitudinal research could further clarify the relationship between loneliness and nomophobia, providing more evidence for either the CIUT or the Displacement Hypothesis.

